# Strengthening approaches to respond to the social and emotional well-being needs of Aboriginal and Torres Strait Islander people: the Cultural Pathways Program

**DOI:** 10.1017/S1463423621000402

**Published:** 2021-06-29

**Authors:** Tina Brodie, Odette Pearson, Luke Cantley, Peita Cooper, Seth Westhead, Alex Brown, Natasha J Howard

**Affiliations:** 1 Wardliparingga Aboriginal Health Equity, South Australian Health and Medical Research Institute (SAHMRI), Adelaide, SA, Australia; 2 Faculty of Health and Medical Sciences, University of Adelaide, Adelaide, SA, Australia; 3 Social Work Innovation Research Living Space, College of Education, Psychology & Social Work, Flinders University, South Australia

**Keywords:** Aboriginal and Torres Strait Islander, case management, evidence-based practice, primary health care, social and emotional well-being, social determinants of health

## Abstract

Aboriginal and Torres Strait Islander holistic health represents the interconnection of social, emotional, spiritual and cultural factors on health and well-being. Social factors (education, employment, housing, transport, food and financial security) are internationally described and recognised as the social determinants of health. The social determinants of health are estimated to contribute to 34% of the overall burden of disease experienced by Aboriginal and Torres Strait Islander people. Primary health care services currently ‘do what it takes’ to address social and emotional well-being needs, including the social determinants of health, and require culturally relevant tools and processes for implementing coordinated and holistic responses. Drawing upon a research-setting pilot program, this manuscript outlines key elements encapsulating a strengths-based approach aimed at addressing Aboriginal and Torres Strait Islander holistic social and emotional well-being.

The Cultural Pathways Program is a response to community identified needs, designed and led by Aboriginal and Torres Strait Islander people and informed by holistic views of health. The program aims to identify holistic needs of Aboriginal and Torres Strait Islander people as the starting point to act on the social determinants of health. Facilitators implement strengths-based practice to identify social and cultural needs (e.g. cultural and community connection, food and financial security, housing, mental health, transport), engage in a goal setting process and broker connections with social and health services. An integrated culturally appropriate clinical supervision model enhances delivery of the program through reflective practice and shared decision making. These embedded approaches enable continuous review and improvement from a program and participant perspective. A developmental evaluation underpins program implementation and the proposed culturally relevant elements could be further tailored for delivery within primary health care services as part of routine care to strengthen systematic identification and response to social and emotional well-being needs.

## Introduction

Aboriginal and Torres Strait Islander knowledge and wisdom has long recognised the role of social and cultural factors on health and well-being (Bartlett & Boffa, 2001). Aboriginal and Torres Strait Islander holistic health philosophy describes social and emotional well-being as the interconnection of social emotional, spiritual, cultural factors on health and wellness of not just individuals but communities (NAHSWP, 1989). Social and emotional well-being as conceptualised by Gee *et al.* (Dudgeon *et al.*, 2014) recognises the ongoing influence of historical, political and social factors on health and social outcomes (Swan & Raphael, 1995; Raphael & Swan, 1997; Dudgeon *et al.*, 2014; Paradies *et al.*, 2015). These social factors (employment, education, housing, income and transport) are internationally described and recognised as the social determinants of health and are estimated to contribute to 34% of the overall burden of disease experienced by Aboriginal and Torres Strait Islander people (ABS, 2013). Both internationally and cross-culturally peer-reviewed literature has established associations, explored pathways and biological mechanisms providing a critical knowledge base on the role of social factors on health (Braveman *et al.*, 2011). Despite these understandings, there is limited evidence on effective intervention strategies that address how these social factors influence health outcomes within the population (Bambra *et al.*, 2010; Thornton *et al.*, 2016; Alegría *et al.*, 2018; Luchenski *et al.*, 2018).

Recent government consultations highlight the importance of self-determined and timely action on the social determinants of health for Aboriginal and Torres Strait Islander communities incorporating system responses that are coordinated, culturally relevant and strengths-based (Andermann, 2016; Commonwealth of Australia, 2017; Frier *et al.*, 2020; Osborne *et al.*, 2013). Health systems face challenges in responding to the complex nature of the social determinants of health with collaborations required across health and social services; nonetheless, the clinical frontline workforce have been recognised as a potential catalyst for change in any systems response (Andermann, 2016). Clinical workforce approaches that include screening clients for social and emotional well-being (which include the social determinants of health) facilitate the early identification and management of needs, planned and coordinated responses and the monitoring of progress and outcomes (Langham *et al.*, 2017).

In a current context, Aboriginal Community Controlled Health Services (ACCHOs) and primary health care services are ‘doing whatever it takes’ to meet the social and emotional well-being needs of Aboriginal and Torres Strait Islander people which includes addressing the social determinants of health in service delivery (CREATE, 2020). Consultations with ACCHOs have highlighted key principles which inform holistic approaches to the social determinants of health including self-determination, accessible and culturally safe care and strong partnerships that support clients to navigate social services (CREATE, 2020). A recent document analysis of 67 ACCHO annual reports found that all services were working to improve clients’ intermediary social determinants of health, specifically material circumstances, biological, behavioural and psychosocial factors (Pearson *et al.*, 2020). Whilst structured and funded Aboriginal and Torres Strait Islander health assessments for preventative care are widely implemented, these assessments are limited by a biomedical focus that inadequately addresses social and cultural factors (Bailie *et al.*, 2019). Across organisations there are varied responses depending on the capacity (i.e. workforce, skills, training and resources) of the primary health care service (CREATE, 2020; Andermann, 2018). Furthermore, service delivery protocols for addressing the social determinants of health and more broadly data systems for monitoring their actions are not well established (Golembiewski *et al.*, 2019; Osborne *et al.*, 2013).

Strengths-based, person centred and empowerment approaches are often used synonymously to describe the delivery of health care for Aboriginal and Torres Strait Islander people. These approaches promote individuals control over their own lives and focus on abilities and resources to enable self-determination (Bovill *et al.*, 2019; Gibson *et al.*, 2020; Saleebey, 1996). Aboriginal and Torres Strait Islander people who have increased control and mastery over their lived experiences are empowered in their engagement with social and health services (Tsey *et al.*, 2010). Health care services commonly describe intentions to deliver strengths-based approaches, yet the practical and genuine implementation with Aboriginal and Torres Strait Islander people is still emerging in practice (Askew *et al.*, 2020; Gibson *et al.*, 2020). Holistic case management models are well suited for strengths-based practice which focuses on empowering people to take charge of their own lives and to support the identification of existing strengths and resources (Saleebey, 1996). Case management approaches whilst diverse across disciplines and in different contexts usually include the following core functions; assessment, planning, linking, monitoring, advocacy and outreach services (Huber, 2002). Case management approaches in primary health care with Aboriginal and Torres Strait Islander people report improvements in self-rated health status, reduction in depression and improved measures of diabetes control (Askew *et al.*, 2015). These findings suggest that patient-led case management has the potential to enhance holistic approaches to social and emotional well-being (Askew *et al.*, 2015).

The effects of colonisation and the continuing social and political oppression and dispossession of Aboriginal and Torres Strait Islander communities have contributed to significant socio-economic and health inequities (Gracey & King, 2009). Persistent and disproportionate inequalities experienced by Aboriginal and Torres Strait Islander people highlight the need to better understand and respond to social and emotional well-being needs which includes the social determinants of health. There is a pressing need for coordinated best practice responses to social and emotional well-being screening and management, dedicated resources, training and ongoing monitoring (Langham *et al.*, 2017). Existing evidence has not yet described approaches that collectively inform health care responses for Aboriginal and Torres Strait Islander social and emotional well-being. To address this gap, a pilot program has been designed within a research setting and includes the following key elements: i) identifying unmet needs, ii) strengths-based case management, iii) document and monitoring, iv) culturally relevant supervision and v) evaluation. The aim of this manuscript is to describe and critically explore the program’s key elements from an Aboriginal and Torres Strait Islander perspective as part of strengthening practice-based evidence on social and emotional well-being.

## Discussion

### Program context

The Cultural Pathways program is implemented by Wardliparingga Aboriginal Health Equity research team in the South Australian Health and Medical Research Institute, Adelaide, South Australia. Wardliparingga undertakes research that is of relevance to South Australian Aboriginal and Torres Strait Islander communities through partnerships, collaboration, respect, reciprocity and for the benefit of community (SAHMRI, 2014). The Cultural Pathways Program is designed and implemented by Aboriginal and Torres Strait Islander people as a response to community identified needs. The program is implemented within an Indigenous methodological framework and from inception to implementation the program has been underpinned by Aboriginal and Torres Strait Islander ways of knowing, being and doing (Rigney, 1999; Martin & Mirraboopa, 2003; Saunders *et al.*, 2010; Wilson, 2011; Smith, 2012). Priority areas for research were established through extensive consultation and engagement with the community (King & Brown, 2015). All programs of work implemented by Wardliparingga have Aboriginal and Torres Strait Islander leadership and governance, through these structures the community consistently highlighted that more holistic responses, which included the social determinants of health, were required. The research team is predominantly Aboriginal and Torres Strait Islander researchers who bring wisdom and experience to the development of the program approach and implementation ensuring consistent alignment with Aboriginal and Torres Strait Islander ways of knowing, being and doing. The program described in this manuscript was approved by the Aboriginal Health Research Ethics Committee of South Australia (AHREC-04-17-733).

The program approach includes comprehensive screening utilising a specifically developed holistic screening tool to identify unmet social and emotional well-being needs. Following screening, facilitators implement strengths-based case management through goal setting, prioritisation and brokering connections to services. program structures embed documentation and monitoring of the program’s social and emotional well-being responses, actions taken to address needs and outcomes for participants. These elements are underpinned by culturally relevant supervision, reflective practice and evaluation. The program approach critically explores the benefits, cultural relevance and responsiveness of common practices in case management. Through a combined understanding of these approaches, the program seeks to inform the evidence base for strengthened and coordinated responses to Aboriginal and Torres Strait Islander social and emotional well-being.

**Figure 1.**
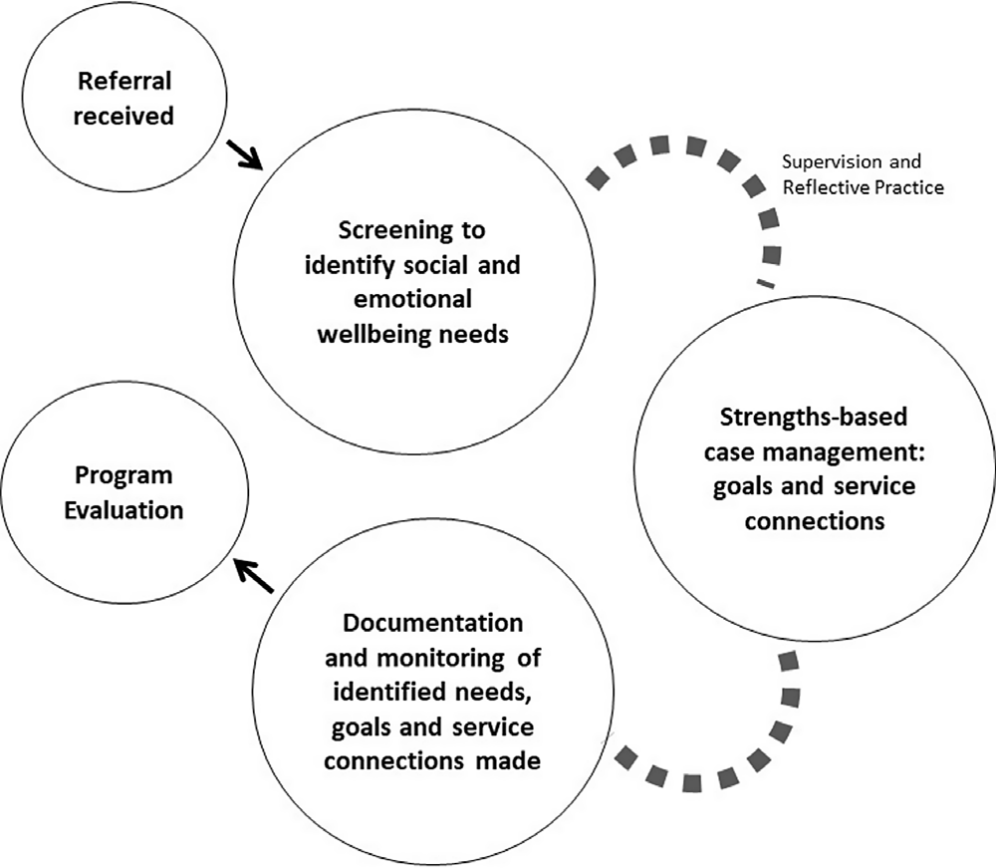
Cultural Pathways Program elements for responding to Aboriginal and Torres Strait Islander social and emotional well-being.

Program delivery is undertaken by male and female facilitators with workforce roles informed by a navigator approach, to assist individuals’ engagement with the health care system and to overcome any barriers to care (Bernardes *et al.*, 2018; Henderson & Kendall, 2011; Whop *et al.*, 2012). Referrals are received from a large-scale population-based biomedical cohort study of Aboriginal and Torres Strait Islander South Australians. As part of the study, all participants receive a comprehensive health assessment that includes questions regarding their social and emotional well-being. Further to this, community engagement and consultations highlighted that post-study follow-up responses for participants would require addressing social and emotional well-being needs such as psychosocial health, financial literacy, food security and material circumstances. Participants are offered a referral to the Cultural Pathways Program, if unmet social and cultural needs are identified during the assessment. The implementation setting replicates real-world service delivery models where presentation may initially be for a physical health need. Upon receipt of referrals from the study team, the Cultural Pathways Program facilitators connect with participants and implement the flexible participant led case management process (Figure 1).

Program elements informing a social and emotional well-being response have been detailed within the following sections, providing the theoretical underpinnings, Cultural Pathways Program approach, embedded Aboriginal and Torres Strait Islander ways of working and opportunities for strengthening practice.

### Identifying unmet needs

Screening and assessment is a common first point of engagement in health settings and appropriate screening delivered as part of routine practice can enhance the timely and effective identification of needs and accordingly inform responses or prompt a more comprehensive assessment (Andermann, 2018). Indigenous specific health assessments are associated with improved preventive care for a range of health needs; however, a greater focus is needed on social and cultural factors (Bailie *et al.*, 2019; Langham *et al.*, 2017). Cultural Pathways Program facilitators implement a modified Social Needs Screening Tool (Health Leads 2016) to identify unmet social needs of participants. Developed through an Aboriginal and Torres Strait Islander researcher led process, with community input to ensure cultural relevance and responsiveness, the adapted holistic tool covers well-being domains including mental health and cultural and community connection and social domains including financial and food security, transport, employment, housing and social isolation. The process of cultural development ensures the questions are relevant, asked the right way, with cultural meaning and are best able to identify the unique needs of Aboriginal and Torres Strait Islander participants (Brown *et al.*, 2013; Langham *et al.*, 2017). Screening processes for the social determinants must be accompanied by plans for action (Gottlieb *et al.*, 2016; Davidson & McGinn, 2019), and as part of the program’s case management approach the screening process assists the Facilitator to understand participant needs and enables the identification and prioritisation of participant goals. By implementing a structured and consistent approach, identifying and documenting unmet needs enable the measurement of actions, activities and the monitoring of participant outcomes.

### Strengths-Based case management

The program’s case management approach includes goal setting, prioritisation and brokering connections to services. Facilitators work in partnership with participants and tailor responses to individual circumstances and needs. A strengths-based approach to case management ensures facilitators focus on clients’ abilities, talents and resources to enable client’s self-determination skills, develop resilience and the ability to respond or navigate similar situations in the future (Saleebey, 1996). Goal setting is a common step in the case of management process (Kisthardt *et al.*, 1992) with theoretical concepts highlighting the importance of collaboration for effective goal setting (Vanpuymbrouck, 2014). An individuals’ sense of control and autonomy influence their willingness to set goals and efforts for achieving them (Vieira & Grantham, 2011). The Australian Integrated Mental Health Initiative (AIMhi) is an existing framework that uses strengths based story telling (Nagel & Thompson, 2007). The Cultural Pathways Program implements a goal and priority setting framework utilising the AIMhi Pictorial Care Plan (Menzies School of Health Research, 2020) to explore physical, emotional, spiritual, cultural, family, social and work contexts to identify worries, strengths and resources. Consistent with Aboriginal and Torres Strait Islander ways of working, facilitators work in partnership with participants to identify and prioritise issues of most importance that will support improved well-being. As part of the strengths-based, empowerment and person-centred approaches, participants define their own priorities contributing to enhanced autonomy, control and self-efficacy.

As part of the ‘brokering’ approach to case management, facilitators connect participants with services to meet their needs. Making a referral to other services, organisations or agencies are widely implemented in health and social services. Social and emotional well-being and social determinants of health needs span across sectors with often multiple services and agencies involved, this requires coordination to minimise the burden on service users and to enable referrals and connections (Kowanko *et al.*, 2009). Brokering connections relies on relationships, understandings of what is available across the breadth of health and social needs and understandings of culturally relevant services (McKenna *et al.*, 2015; Treatment Center for Substance Abuse, 2000). To support this approach, facilitators undertake service mapping exercises to identify the available services and will pro-actively seek the most appropriate service to connect a participant to and reduce barriers to access these services (Huber, 2002). Facilitators actively support participants to access services by contacting services on behalf of participants, supporting participants when they contact services themselves and follow up contact with participants to monitor progress. If necessary, facilitators address any challenges or barriers to support the best possible outcome. The active and coordinated approach to brokering connections enhances service access for participants and enables the program to also monitor brokerage outcomes.

### Documentation and monitoring

Program monitoring involves measuring and reporting on progress and creates opportunities for continuous quality improvement (Hudson, 2016). Currently, health services rarely systematically collect data about or measure activity on the social determinants of health and require a mechanism to monitor and evaluate the impact of social and emotional well-being services they provide to address health outcomes (Langham *et al.*, 2017; Reeve *et al.*, 2015). Comprehensive understandings of the most appropriate measures for Aboriginal and Torres Strait Islander social and emotional well-being and the social determinants of health are still emerging. Existing national measures of well-being include psychological distress, positive well-being, anger, life stressors, discrimination, cultural identification and removal from natural family (AIHW, 2009). Measures for the social determinants of health as described by the World Health Organization (WHO) Conceptual Framework (Solar & Irwin, 2010) and outlined in the Aboriginal and Torres Strait Islander Health Performance Framework (AHMAC, 2017) include domains such as connection to country, education, employment, health system, housing, income and transport.

The Cultural Pathways Program combines social and emotional well-being and social determinants of health measures as part of the programs’ monitoring framework. The program utilises REDCap (Research Electronic Data Capture), a secure web platform for managing online databases (Harris *et al.*, 2009). The platform collects participant information, demographics and activity data which include when and how people are contacted and the services provided by social/health domain. The program measures factors such as unmet needs, identified goals, whether they have been achieved and the service connections made. The program utilises routine data for ongoing monitoring, quality improvement and as part of funding requirements and obligations. The data collected by the program was informed by Aboriginal and Torres Strait Islander understandings of health and well-being and the wisdom and expertise of the research team and community. The process included the collective development of culturally relevant measures in relation to social and emotional well-being, specifically practical ways to measure progress towards addressing complex social and cultural factors. This process enabled the program to capture information that is useful and relevant for Aboriginal and Torres Strait Islander people. A structured and consistent approach to identifying needs and a specifically designed monitoring framework enables the program to measure progress or outcomes which can be used to understand the needs of service users, to plan responses and to advocate for resources (Harfield *et al.*, 2018).

### Culturally relevant supervision

Reflective practice and clinical supervision are recognised by many professions for their role in supporting enhanced clinical practice as well as the health and well-being of the workforce (Koivu *et al.*, 2012; Scerra, 2012; Thompson & Pascal, 2012). This is particularly important for Aboriginal and Torres Strait Islander health workers and practitioners who have complex experiences including burnout and vicarious trauma (Nelson *et al.*, 2015). The Aboriginal and Torres Strait Islander health workforce and non-Indigenous workers in Aboriginal and Torres Strait Islander health contexts require access to high-quality cultural and clinical supervision which supports cultural safety, improved practice and well-being (Bainbridge *et al.*, 2015; Truong *et al.*, 2014). Available frameworks for culturally appropriate supervision with Aboriginal and Torres Strait Islander people include considerations for working with community, looking after self, understanding of roles and professional practice (Koivu *et al.*, 2012; Nelson *et al.*, 2015; Scerra, 2012; Victorian Aboriginal Child Care Agency (VACCA), 2013; Victorian Dual Diagnosis Education and Training Unit (VDDI), 2012). Despite the important role of culturally relevant clinical supervision in enhanced service delivery and the support and retention of the workforce in health care settings (AHCSA, 2020), evidence-based understanding of applied practice models are still emerging in peer reviewed evidence.

The Cultural Pathways Program utilises these existing frameworks as well as the knowledge and experience of program staff to implement a culturally relevant reflective practice and supervision model. An experienced Aboriginal clinician supports facilitators through a range of structures including weekly clinical yarning, one to one yarning and debriefing opportunities as required. Facilitators share perspectives, feelings, challenges, barriers and enablers in relation to both clinical practice as well as system, policy and organisational factors which impact the participant, Facilitator, or the program. Fundamentally, the supervision and reflective practice model are culturally grounded in relationships and yarning to support the cultural safety for Aboriginal and Torres Strait Islander participants whilst also enabling the retention and well-being of the Aboriginal and Torres Strait Islander workforce.

### Developmental evaluation

Evaluating health programs and initiatives supports implementation across different contexts utilising insights into how and why they work and whether they have been effective (Lokuge *et al.*, 2017). There is an increasing recognition of the important role of evidence-based programs featuring high quality and culturally relevant evaluation (Productivity Commission, 2019). The Cultural Pathways Program is underpinned by an Indigenous methodological evaluation framework which utilises Developmental Evaluation, an approach to evaluation that supports innovation and adaptation in complex environments (Fagen *et al.*, 2011; Patton, 2010), and is consistent with Indigenous methodology and participatory approaches requiring partnerships, trust and shared decision making (Gamble, 2008). The key to developmental evaluation is that the evaluator works with the team in real-time, asking evaluation questions, examining and tracking implications of adaptations and providing timely feedback as the program is implemented and modified or adapted as needed. The evaluator as an Aboriginal woman is immersed in as an insider drawing heavily on reflective practice and utilising the cultural knowledge and expertise of the evaluator as part of the evaluation method. The aim of the evaluation is to understand the process including what was delivered, how it was implemented and the experiences of program participants. The evaluation through reflective and formative methods supports further understanding on the interactions between facilitators, program participants and the broader health and social service contexts. The evaluation framework includes community engagement, governance and approaches which have been purposely selected for their consistency with Indigenous methodologies. This framework ensures that the participation and voice of the community are therefore embedded throughout implementation to support  tangible benefits to the community (SAHMRI, 2014).

## Conclusions

There is a knowledge to action gap on how to assess and address the social determinants of health within clinical practice to inform the development of coordinated, culturally relevant and strength-based responses to meet the holistic social and emotional well-being needs of Aboriginal and Torres Strait Islander people and communities.

Primary health care services, often as the entry point for accessing health services, are well positioned to implement coordinated health equity responses which include addressing the social determinants of health (Pereira *et al.*, 2017; Rasanathan *et al.*, 2011). The absence of a readily applied model creates challenges for the provision of coordinated, resourced and systemic responses to the social determinants of health (CREATE, 2020). Routine screening for unmet needs, implementing strengths-based practice, connecting people to what they need, monitoring service provision and providing clinical and cultural support for the Aboriginal and Torres Strait Islander workforce align to existing practice and are transferable across contexts. Continuous quality improvement and monitoring enables primary health care services to embed new practices into services, systems and routines (Gardner *et al.*, 2010).

The ability to implement holistic approaches to Aboriginal and Torres Strait Islander health through the intersection of health and social services requires adequate resources, training and support to clinical workforce (Andermann, 2016), including consideration of roles, responsibilities, scope of practice and readiness to implement strengths-based approaches. These changes cannot be implemented without addressing the ongoing impacts of racism and oppression of Aboriginal and Torres Strait Islander people, allowing for culturally safe systems which are able to meet holistic social and emotional well-being needs (Curtis *et al.*, 2019; Durey, 2010; Laverty *et al.*, 2017; Muise, 2019; Secombe *et al.*, 2019).

The Cultural Pathways Program builds on existing approaches to contribute to practice-based evidence of culturally relevant case management approaches which can be utilised as part of routine care to strengthen the systematic identification and response in primary health care delivery. The combined understandings of the elements outlined in this manuscript provide a framework to inform service planning and tailored implementation which can strengthen social and emotional well-being responses for Aboriginal and Torres Strait Islander people.
